# 2-(4-Bromo­benzo­yl)-1-ferrocenyl­spiro­[11*H*-pyrrolidizine-3,11′-indeno­[1,2-*b*]quinoxaline]

**DOI:** 10.1107/S1600536813023064

**Published:** 2013-08-23

**Authors:** Sivasubramanian Suhitha, Krishnaswamy Gunasekaran, Adukamparai Rajukrishnan Sureshbabu, Raghavachary Raghunathan, Devadasan Velmurugan

**Affiliations:** a Centre of Advanced Study in Crystallography and Biophysics, University of Madras, Guindy Campus, Chennai 600 025, India; b Department of Organic Chemistry, University of Madras, Guindy Campus, Chennai 600 025, India

## Abstract

In the title compound, [Fe(C_5_H_5_)(C_33_H_25_BrN_3_O)], the fused four-ring system, 11*H*-indeno­[1,2-*b*]quinoxaline is essentially planar, with a maximum deviation of 0.087 (3) Å from the least-squares plane of the attached benzene ring. The pyrrolidine rings adopt envelope conformation and make a dihedral angle of 51.76 (19)° with each other. The cyclopentadiene rings of the ferrocenyl moiety have an eclipsed conformation. The Br atom deviates by 0.0190 (9) Å from the attached benzene ring. The mol­ecular structure features an intra­molecular C—H⋯N inter­action, which generates an *S*(8) ring motif. The crystal packing features C—H⋯O inter­actions, which generate *R*_2_^2^(18) centrosymmetric dimers, as well as C—H⋯π inter­actions.

## Related literature

For the biological activity of ferrocene derivatives, see: Jaouen *et al.* (2004 ▶); Biot *et al.* (2004 ▶); Fouda *et al.* (2007 ▶). For a related structure, see: Vijayakumar *et al.* (2012 ▶). For graph-set notation, see: Bernstein *et al.* (1995 ▶).
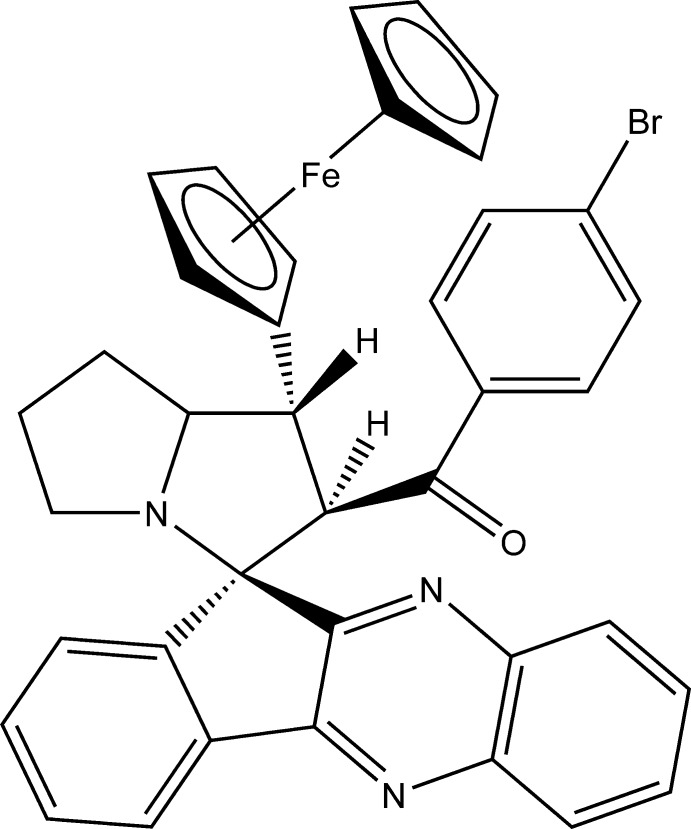


## Experimental

### 

#### Crystal data

[Fe(C_5_H_5_)(C_33_H_25_BrN_3_O)]
*M* *_r_* = 680.41
Triclinic, 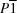

*a* = 9.3668 (3) Å
*b* = 9.9785 (3) Å
*c* = 18.6303 (5) Å
α = 89.724 (1)°
β = 75.967 (1)°
γ = 63.236 (1)°
*V* = 1497.37 (8) Å^3^
*Z* = 2
Mo *K*α radiation
μ = 1.88 mm^−1^
*T* = 293 K
0.30 × 0.25 × 0.20 mm

#### Data collection

Bruker SMART APEXII area-detector diffractometer
Absorption correction: multi-scan (*SADABS*; Bruker, 2008 ▶) *T*_min_ = 0.603, *T*_max_ = 0.706
22634 measured reflections
6177 independent reflections
5078 reflections with *I* > 2σ(*I*)
*R*_int_ = 0.028

#### Refinement

*R*[*F* ^2^ > 2σ(*F* ^2^)] = 0.053
*wR*(*F* ^2^) = 0.137
*S* = 1.04
6177 reflections
397 parameters
H-atom parameters constrained
Δρ_max_ = 1.87 e Å^−3^
Δρ_min_ = −1.63 e Å^−3^

### 

Data collection: *APEX2* (Bruker, 2008 ▶); cell refinement: *SAINT* (Bruker, 2008 ▶); data reduction: *SAINT*; program(s) used to solve structure: *SHELXS97* (Sheldrick, 2008 ▶); program(s) used to refine structure: *SHELXL97* (Sheldrick, 2008 ▶); molecular graphics: *ORTEP-3 for Windows* (Farrugia, 2012 ▶); software used to prepare material for publication: *SHELXL97* and *PLATON* (Spek, 2009 ▶).

## Supplementary Material

Crystal structure: contains datablock(s) global, I. DOI: 10.1107/S1600536813023064/pv2643sup1.cif

Structure factors: contains datablock(s) I. DOI: 10.1107/S1600536813023064/pv2643Isup2.hkl

Additional supplementary materials:  crystallographic information; 3D view; checkCIF report

## Figures and Tables

**Table 1:** Hydrogen-bond geometry (Å, °) *Cg*1, *Cg*2 and *Cg*3 are the centroids of the C29–C33, C9–C15 and N1/N2/C1/C6/C7/C11 rings, respectively.

*D*—H⋯*A*	*D*—H	H⋯*A*	*D*⋯*A*	*D*—H⋯*A*
C28—H28⋯N2	0.93	2.56	3.358 (5)	145
C13—H13⋯O1^i^	0.93	2.58	3.318 (4)	137
C4—H4⋯*Cg*1^ii^	0.93	2.69	3.580 (4)	159
C17—H17*B*⋯*Cg*2^iii^	0.97	2.84	3.743 (4)	155
C36—H36⋯*Cg*3^iv^	0.98	2.70	3.486 (5)	137

Symmetry codes: (i) 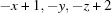; (ii) 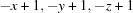; (iii) 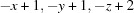; (iv) 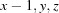.

**Fig. 1.**
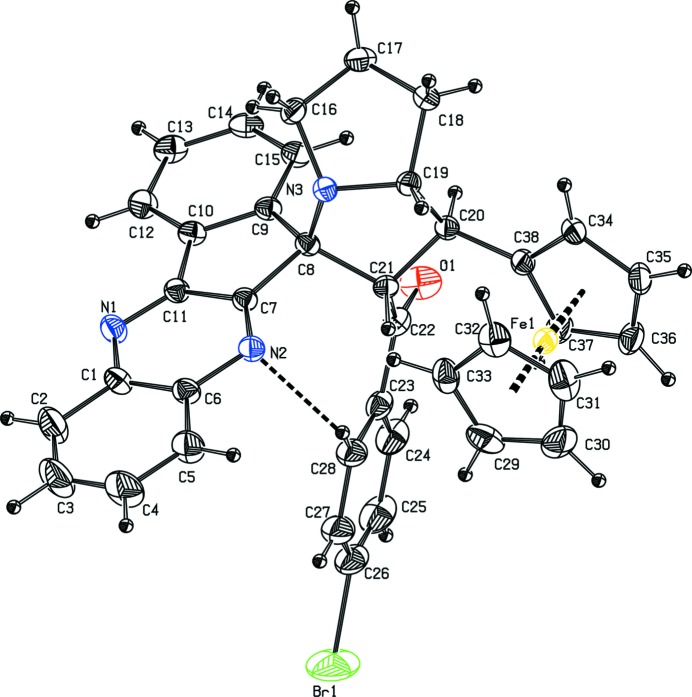
The molecular structure of the title compound, showing displacement ellipsoids drawn at the 30% probability level.

**Fig. 2.**
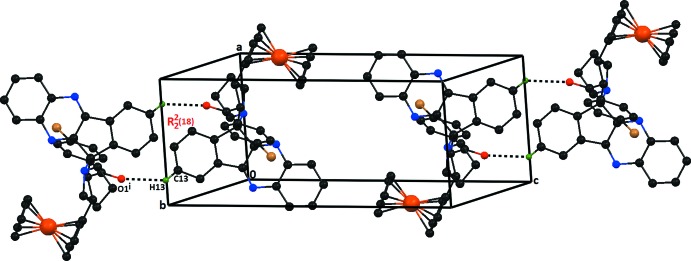
The crystal packing of the title compound viewed down the b axis. H-atoms not involved in H-bonds have been excluded for clarity.

## supplementary crystallographic information

### 1. Comment

Biological activities like antimalarial, antifungal (Biot *et al.*, 2004), antitumor (Jaouen *et al.*, 2004), and antibacterial (Fouda *et al.*, 2007) are well known for ferrocene containing compounds. The information of molecular conformation and crystal packing of the title compound were obtained and analyzed using X-ray diffraction study against this background.

In the title compound, the four fused ring system, 11H-indeno[1,2-b]quinoxaline is essentially planar with a maximum deviation of -0.087 (3) Å for C8 atom. The pyrrolidine ring (N3/C16–C19) adopts a C17-*envelope* conformation with C17 -0.230 (4) Å out of the mean plane formed by the remaining ring atoms. The pyrrolidine ring (N3/C8/C19–C21) adopts a C21-*envelope* conformation with C21 -0.211 (3) Å out of the mean plane formed by the remaining ring atoms; the pyrrolidine rings make a dihedral angle of 51.76 (19)° with each other. The pyrrolidine ring (N3/C8/C19/C20/C21) makes a dihedral angle of 87.40 (18)° with the cyclopentane ring (C7-C11) which shows they are almost orthogonal to each other. The dihedral angle between the cp rings in the ferrocene moiety is 4.8 (2) °, which shows that they are almost coplanar to each other. The bromine atom Br1 attached with the benzenel ring (C23-C28) deviates by 0.0190 (9)Å.

The molecular structure is stabilized by C28—H28···N2 intramolecular interaction, which generates a *S*(8) ring motif (Fig. 1). The crystal packing is stabilized by inter-molecular C13—H13···O1^i^ interactions, which generate *R*^2^_2_(18) centrosymmetric dimers (Bernstein *et al.*, 1995) (Fig. 2). The crystal packing is further stabilized by inter-molecular C4—H4···Cg1, C17—H17B···Cg2 and C36—H36···Cg3 inter-molecular interactions, where Cg1, Cg2 and Cg3 are the centers of gravity of rings (C29–C33), (C9–C15) and (N1/N2/C1/C6/C7/C11), respectively (Table 1).

### 2. Experimental

Ninhydrin (1 mM) and 1, 2-phenylenediamine (1 mM) were mixed and stirred with 10 mL of methanol for 10 min. To this mixture proline (1 mM) and 1-ferrcenyl- 3-(4-bromo)benzoyl-prop-2-ene dipolarophile (1 mM) were added and was refluxed up to the end of the reaction as observed by TLC. The solvent content from the mixture was removed under reduced pressure and the crude product was obtained. Using column chromatography the crude extract was purified by petroleum ether and ethyl acetate (4:1). Finally, single crystals suitable for the X-ray diffraction were obtained by slow evaporation at room temperature.

### 3. Refinement

Hydrogen atoms were placed in calculated positions with C—H ranging from 0.93Å to 0.98Å and refined using the riding model approximation with a fixed isotropic displacement parameter *U*_iso_(H) = 1.2 *U*_eq_(C).

### Crystal data

**Table:**

[Fe(C_5_H_5_)(C_33_H_25_BrN_3_O)]	*Z* = 2
*M**_r_* = 680.41	*F*(000) = 696
Triclinic, *P*1	*D*_x_ = 1.509 Mg m^−^^3^
Hall symbol: -P 1	Mo *K*α radiation, λ = 0.71073 Å
*a* = 9.3668 (3) Å	Cell parameters from 6177 reflections
*b* = 9.9785 (3) Å	θ = 1.1–26.5°
*c* = 18.6303 (5) Å	µ = 1.88 mm^−^^1^
α = 89.724 (1)°	*T* = 293 K
β = 75.967 (1)°	Block, colourless
γ = 63.236 (1)°	0.30 × 0.25 × 0.20 mm
*V* = 1497.37 (8) Å^3^	

### Data collection

**Table:**

Bruker SMART APEXII area-detector diffractometer	6177 independent reflections
Radiation source: fine-focus sealed tube	5078 reflections with *I* > 2σ(*I*)
Graphite monochromator	*R*_int_ = 0.028
ω and φ scans	θ_max_ = 26.5°, θ_min_ = 1.1°
Absorption correction: multi-scan (*SADABS*; Bruker, 2008)	*h* = −10→11
*T*_min_ = 0.603, *T*_max_ = 0.706	*k* = −12→12
22634 measured reflections	*l* = −23→22

### Refinement

**Table:**

Refinement on *F*^2^	Primary atom site location: structure-invariant direct methods
Least-squares matrix: full	Secondary atom site location: difference Fourier map
*R*[*F*^2^ > 2σ(*F*^2^)] = 0.053	Hydrogen site location: inferred from neighbouring sites
*wR*(*F*^2^) = 0.137	H-atom parameters constrained
*S* = 1.04	*w* = 1/[σ^2^(*F*_o_^2^) + (0.0539*P*)^2^ + 2.514*P*] where *P* = (*F*_o_^2^ + 2*F*_c_^2^)/3
6177 reflections	(Δ/σ)_max_ = 0.001
397 parameters	Δρ_max_ = 1.87 e Å^−^^3^
0 restraints	Δρ_min_ = −1.63 e Å^−^^3^

### Special details

**Table:**

Geometry. All esds (except the esd in the dihedral angle between two l.s. planes) are estimated using the full covariance matrix. The cell esds are taken into account individually in the estimation of esds in distances, angles and torsion angles; correlations between esds in cell parameters are only used when they are defined by crystal symmetry. An approximate (isotropic) treatment of cell esds is used for estimating esds involving l.s. planes.
Refinement. Refinement of F^2^ against ALL reflections. The weighted R-factor wR and goodness of fit S are based on F^2^, conventional R-factors R are based on F, with F set to zero for negative F^2^. The threshold expression of F^2^ > 2sigma(F^2^) is used only for calculating R-factors(gt) etc. and is not relevant to the choice of reflections for refinement. R-factors based on F^2^ are statistically about twice as large as those based on F, and R- factors based on ALL data will be even larger.

### Fractional atomic coordinates and isotropic or equivalent isotropic displacement parameters (Å^2^)

**Table:**

	*x*	*y*	*z*	*U*_iso_*/*U*_eq_	
C1	0.8769 (4)	0.1667 (4)	0.66122 (18)	0.0415 (7)	
C2	1.0270 (5)	0.1122 (5)	0.6044 (2)	0.0648 (11)	
H2	1.1266	0.0461	0.6141	0.078*	
C3	1.0257 (6)	0.1565 (6)	0.5353 (3)	0.0797 (14)	
H3	1.1250	0.1202	0.4980	0.096*	
C4	0.8782 (6)	0.2554 (6)	0.5196 (2)	0.0740 (13)	
H4	0.8799	0.2839	0.4719	0.089*	
C5	0.7313 (5)	0.3108 (5)	0.5734 (2)	0.0557 (10)	
H5	0.6332	0.3762	0.5622	0.067*	
C6	0.7274 (4)	0.2693 (4)	0.64563 (17)	0.0386 (7)	
C7	0.5854 (4)	0.2820 (3)	0.76391 (15)	0.0304 (6)	
C8	0.4385 (3)	0.3328 (3)	0.83310 (15)	0.0295 (6)	
C9	0.5232 (4)	0.2280 (3)	0.88732 (16)	0.0320 (6)	
C10	0.6945 (4)	0.1452 (3)	0.85599 (16)	0.0343 (6)	
C11	0.7351 (4)	0.1757 (3)	0.77898 (16)	0.0328 (6)	
C12	0.7994 (4)	0.0525 (4)	0.89628 (18)	0.0431 (8)	
H12	0.9131	−0.0019	0.8746	0.052*	
C13	0.7309 (5)	0.0429 (4)	0.96949 (19)	0.0455 (8)	
H13	0.7989	−0.0173	0.9980	0.055*	
C14	0.5617 (4)	0.1226 (4)	1.00030 (17)	0.0407 (7)	
H14	0.5173	0.1141	1.0494	0.049*	
C15	0.4560 (4)	0.2150 (4)	0.96023 (17)	0.0374 (7)	
H15	0.3421	0.2672	0.9819	0.045*	
C16	0.4140 (4)	0.5472 (4)	0.91829 (19)	0.0408 (7)	
H16A	0.4445	0.6268	0.9053	0.049*	
H16B	0.5058	0.4640	0.9311	0.049*	
C17	0.2585 (4)	0.6056 (4)	0.98274 (18)	0.0450 (8)	
H17A	0.2503	0.5238	1.0087	0.054*	
H17B	0.2552	0.6790	1.0179	0.054*	
C18	0.1230 (4)	0.6781 (4)	0.94348 (18)	0.0430 (7)	
H18A	0.1028	0.7804	0.9353	0.052*	
H18B	0.0204	0.6806	0.9724	0.052*	
C19	0.1898 (4)	0.5771 (3)	0.86938 (16)	0.0326 (6)	
H19	0.1620	0.6405	0.8295	0.039*	
C20	0.1343 (3)	0.4550 (3)	0.86635 (16)	0.0317 (6)	
H20	0.1134	0.4242	0.9162	0.038*	
C21	0.2879 (3)	0.3238 (3)	0.81511 (15)	0.0302 (6)	
H21	0.2899	0.3456	0.7636	0.036*	
C22	0.2901 (4)	0.1712 (4)	0.82134 (18)	0.0391 (7)	
C23	0.3566 (4)	0.0614 (4)	0.7524 (2)	0.0424 (7)	
C24	0.3524 (5)	−0.0764 (4)	0.7587 (3)	0.0576 (10)	
H24	0.3103	−0.0989	0.8051	0.069*	
C25	0.4111 (6)	−0.1799 (5)	0.6958 (3)	0.0734 (14)	
H25	0.4095	−0.2722	0.7002	0.088*	
C26	0.4712 (5)	−0.1460 (5)	0.6274 (3)	0.0655 (12)	
C27	0.4777 (5)	−0.0127 (5)	0.6198 (2)	0.0601 (10)	
H27	0.5201	0.0085	0.5731	0.072*	
C28	0.4208 (4)	0.0906 (4)	0.6822 (2)	0.0484 (8)	
H28	0.4255	0.1814	0.6770	0.058*	
C29	0.0989 (6)	0.4878 (5)	0.6368 (2)	0.0650 (11)	
H29	0.1603	0.3793	0.6194	0.078*	
C30	−0.0631 (6)	0.5873 (7)	0.6318 (2)	0.0728 (14)	
H30	−0.1334	0.5605	0.6100	0.087*	
C31	−0.1036 (5)	0.7318 (6)	0.6630 (2)	0.0672 (13)	
H31	−0.2081	0.8237	0.6672	0.081*	
C32	0.0316 (5)	0.7225 (5)	0.6868 (2)	0.0536 (9)	
H32	0.0373	0.8064	0.7108	0.064*	
C33	0.1567 (4)	0.5718 (5)	0.6704 (2)	0.0511 (9)	
H33	0.2648	0.5321	0.6812	0.061*	
C34	−0.1484 (4)	0.6599 (4)	0.85115 (17)	0.0405 (7)	
H34	−0.1491	0.7473	0.8754	0.049*	
C35	−0.2744 (4)	0.6630 (5)	0.82032 (19)	0.0484 (8)	
H35	−0.3776	0.7529	0.8201	0.058*	
C36	−0.2267 (4)	0.5159 (5)	0.7909 (2)	0.0518 (9)	
H36	−0.2904	0.4851	0.7662	0.062*	
C37	−0.0698 (4)	0.4184 (4)	0.80284 (19)	0.0435 (8)	
H37	−0.0061	0.3093	0.7873	0.052*	
C38	−0.0201 (4)	0.5077 (4)	0.84020 (16)	0.0348 (6)	
N1	0.8802 (3)	0.1180 (3)	0.73019 (15)	0.0420 (6)	
N2	0.5773 (3)	0.3290 (3)	0.69911 (14)	0.0356 (6)	
N3	0.3720 (3)	0.4961 (3)	0.85602 (13)	0.0321 (5)	
O1	0.2360 (4)	0.1392 (3)	0.88107 (15)	0.0613 (7)	
Fe1	−0.05693 (5)	0.58417 (5)	0.74004 (2)	0.03596 (14)	
Br1	0.54872 (10)	−0.28490 (7)	0.54137 (4)	0.1181 (3)	

### Atomic displacement parameters (Å^2^)

**Table:**

	*U*^11^	*U*^22^	*U*^33^	*U*^12^	*U*^13^	*U*^23^
C1	0.0385 (17)	0.0447 (18)	0.0328 (16)	−0.0166 (14)	−0.0003 (13)	0.0003 (13)
C2	0.041 (2)	0.077 (3)	0.052 (2)	−0.014 (2)	0.0034 (17)	0.004 (2)
C3	0.059 (3)	0.103 (4)	0.049 (2)	−0.028 (3)	0.015 (2)	0.006 (2)
C4	0.077 (3)	0.099 (4)	0.037 (2)	−0.040 (3)	0.001 (2)	0.017 (2)
C5	0.056 (2)	0.073 (3)	0.0352 (18)	−0.029 (2)	−0.0085 (16)	0.0162 (17)
C6	0.0418 (17)	0.0453 (18)	0.0287 (15)	−0.0228 (15)	−0.0043 (13)	0.0044 (13)
C7	0.0322 (14)	0.0306 (14)	0.0272 (14)	−0.0135 (12)	−0.0080 (11)	0.0044 (11)
C8	0.0308 (14)	0.0293 (14)	0.0249 (13)	−0.0113 (12)	−0.0067 (11)	0.0045 (11)
C9	0.0375 (15)	0.0304 (14)	0.0270 (14)	−0.0139 (12)	−0.0104 (12)	0.0049 (11)
C10	0.0399 (16)	0.0298 (15)	0.0304 (15)	−0.0128 (13)	−0.0114 (12)	0.0050 (12)
C11	0.0326 (15)	0.0297 (14)	0.0319 (15)	−0.0106 (12)	−0.0094 (12)	0.0022 (11)
C12	0.0425 (18)	0.0355 (16)	0.0420 (18)	−0.0073 (14)	−0.0169 (14)	0.0071 (14)
C13	0.059 (2)	0.0363 (17)	0.0393 (18)	−0.0154 (16)	−0.0246 (16)	0.0129 (14)
C14	0.062 (2)	0.0381 (17)	0.0266 (15)	−0.0257 (16)	−0.0135 (14)	0.0096 (12)
C15	0.0429 (17)	0.0361 (16)	0.0315 (15)	−0.0179 (14)	−0.0079 (13)	0.0061 (12)
C16	0.0357 (16)	0.0385 (17)	0.0493 (19)	−0.0153 (14)	−0.0170 (14)	−0.0033 (14)
C17	0.054 (2)	0.0453 (19)	0.0358 (17)	−0.0209 (16)	−0.0158 (15)	−0.0010 (14)
C18	0.0345 (16)	0.0441 (18)	0.0411 (18)	−0.0117 (14)	−0.0070 (13)	−0.0095 (14)
C19	0.0312 (14)	0.0317 (15)	0.0320 (15)	−0.0105 (12)	−0.0119 (12)	0.0032 (12)
C20	0.0309 (14)	0.0382 (15)	0.0250 (14)	−0.0153 (13)	−0.0069 (11)	0.0055 (11)
C21	0.0333 (14)	0.0333 (15)	0.0249 (13)	−0.0154 (12)	−0.0094 (11)	0.0052 (11)
C22	0.0422 (17)	0.0375 (16)	0.0422 (18)	−0.0194 (14)	−0.0175 (14)	0.0110 (14)
C23	0.0390 (17)	0.0322 (16)	0.059 (2)	−0.0137 (14)	−0.0234 (15)	0.0039 (14)
C24	0.060 (2)	0.0398 (19)	0.082 (3)	−0.0240 (18)	−0.033 (2)	0.0107 (19)
C25	0.075 (3)	0.035 (2)	0.119 (4)	−0.022 (2)	−0.049 (3)	−0.002 (2)
C26	0.058 (2)	0.045 (2)	0.084 (3)	−0.0070 (19)	−0.035 (2)	−0.018 (2)
C27	0.057 (2)	0.053 (2)	0.062 (2)	−0.0159 (19)	−0.0194 (19)	−0.0120 (18)
C28	0.050 (2)	0.0398 (18)	0.053 (2)	−0.0180 (16)	−0.0156 (16)	−0.0040 (15)
C29	0.071 (3)	0.074 (3)	0.0335 (19)	−0.028 (2)	0.0031 (18)	−0.0028 (18)
C30	0.074 (3)	0.123 (4)	0.036 (2)	−0.053 (3)	−0.025 (2)	0.017 (2)
C31	0.048 (2)	0.087 (3)	0.052 (2)	−0.017 (2)	−0.0178 (18)	0.035 (2)
C32	0.055 (2)	0.059 (2)	0.048 (2)	−0.0292 (19)	−0.0099 (17)	0.0200 (17)
C33	0.0373 (17)	0.070 (2)	0.0415 (19)	−0.0228 (18)	−0.0069 (14)	0.0207 (17)
C34	0.0303 (15)	0.0507 (19)	0.0332 (16)	−0.0146 (14)	−0.0045 (12)	0.0004 (14)
C35	0.0277 (15)	0.068 (2)	0.0429 (18)	−0.0174 (16)	−0.0082 (13)	0.0077 (17)
C36	0.0408 (18)	0.076 (3)	0.054 (2)	−0.0379 (19)	−0.0158 (16)	0.0109 (19)
C37	0.0438 (18)	0.051 (2)	0.0462 (19)	−0.0301 (16)	−0.0140 (15)	0.0101 (15)
C38	0.0308 (14)	0.0470 (18)	0.0276 (14)	−0.0203 (13)	−0.0054 (11)	0.0063 (12)
N1	0.0332 (13)	0.0411 (15)	0.0395 (15)	−0.0088 (12)	−0.0056 (11)	0.0018 (12)
N2	0.0357 (13)	0.0417 (14)	0.0285 (13)	−0.0173 (11)	−0.0084 (10)	0.0072 (11)
N3	0.0302 (12)	0.0313 (12)	0.0325 (13)	−0.0122 (10)	−0.0085 (10)	0.0023 (10)
O1	0.094 (2)	0.0613 (17)	0.0467 (15)	−0.0507 (16)	−0.0196 (14)	0.0214 (13)
Fe1	0.0312 (2)	0.0472 (3)	0.0305 (2)	−0.0182 (2)	−0.00988 (17)	0.00543 (18)
Br1	0.1451 (6)	0.0685 (4)	0.1205 (5)	−0.0226 (4)	−0.0544 (5)	−0.0415 (3)

### Geometric parameters (Å, º)

**Table:**

C1—N1	1.374 (4)	C20—H20	0.9800
C1—C2	1.410 (5)	C21—C22	1.518 (4)
C1—C6	1.414 (5)	C21—H21	0.9800
C2—C3	1.360 (6)	C22—O1	1.211 (4)
C2—H2	0.9300	C22—C23	1.499 (5)
C3—C4	1.391 (7)	C23—C28	1.390 (5)
C3—H3	0.9300	C23—C24	1.397 (5)
C4—C5	1.362 (6)	C24—C25	1.390 (6)
C4—H4	0.9300	C24—H24	0.9300
C5—C6	1.403 (5)	C25—C26	1.369 (7)
C5—H5	0.9300	C25—H25	0.9300
C6—N2	1.377 (4)	C26—C27	1.364 (6)
C7—N2	1.300 (4)	C26—Br1	1.889 (4)
C7—C11	1.421 (4)	C27—C28	1.383 (5)
C7—C8	1.526 (4)	C27—H27	0.9300
C8—N3	1.480 (4)	C28—H28	0.9300
C8—C9	1.541 (4)	C29—C33	1.403 (6)
C8—C21	1.566 (4)	C29—C30	1.417 (7)
C9—C15	1.384 (4)	C29—Fe1	2.037 (4)
C9—C10	1.398 (4)	C29—H29	0.9800
C10—C12	1.385 (4)	C30—C31	1.403 (7)
C10—C11	1.460 (4)	C30—Fe1	2.031 (4)
C11—N1	1.306 (4)	C30—H30	0.9800
C12—C13	1.383 (5)	C31—C32	1.406 (6)
C12—H12	0.9300	C31—Fe1	2.031 (4)
C13—C14	1.379 (5)	C31—H31	0.9800
C13—H13	0.9300	C32—C33	1.404 (6)
C14—C15	1.385 (4)	C32—Fe1	2.048 (4)
C14—H14	0.9300	C32—H32	0.9800
C15—H15	0.9300	C33—Fe1	2.054 (3)
C16—N3	1.472 (4)	C33—H33	0.9800
C16—C17	1.515 (5)	C34—C35	1.422 (5)
C16—H16A	0.9700	C34—C38	1.426 (5)
C16—H16B	0.9700	C34—Fe1	2.040 (3)
C17—C18	1.513 (5)	C34—H34	0.9800
C17—H17A	0.9700	C35—C36	1.398 (6)
C17—H17B	0.9700	C35—Fe1	2.024 (3)
C18—C19	1.529 (4)	C35—H35	0.9800
C18—H18A	0.9700	C36—C37	1.423 (5)
C18—H18B	0.9700	C36—Fe1	2.032 (3)
C19—N3	1.477 (4)	C36—H36	0.9800
C19—C20	1.530 (4)	C37—C38	1.425 (4)
C19—H19	0.9800	C37—Fe1	2.052 (3)
C20—C38	1.503 (4)	C37—H37	0.9800
C20—C21	1.531 (4)	C38—Fe1	2.060 (3)
			
N1—C1—C2	119.0 (3)	C33—C29—H29	125.9
N1—C1—C6	121.8 (3)	C30—C29—H29	125.9
C2—C1—C6	119.2 (3)	Fe1—C29—H29	125.9
C3—C2—C1	119.8 (4)	C31—C30—C29	107.4 (4)
C3—C2—H2	120.1	C31—C30—Fe1	69.8 (2)
C1—C2—H2	120.1	C29—C30—Fe1	69.8 (2)
C2—C3—C4	121.1 (4)	C31—C30—H30	126.3
C2—C3—H3	119.5	C29—C30—H30	126.3
C4—C3—H3	119.5	Fe1—C30—H30	126.3
C5—C4—C3	120.5 (4)	C30—C31—C32	108.4 (4)
C5—C4—H4	119.7	C30—C31—Fe1	69.8 (2)
C3—C4—H4	119.7	C32—C31—Fe1	70.5 (2)
C4—C5—C6	120.2 (4)	C30—C31—H31	125.8
C4—C5—H5	119.9	C32—C31—H31	125.8
C6—C5—H5	119.9	Fe1—C31—H31	125.8
N2—C6—C5	118.9 (3)	C33—C32—C31	108.0 (4)
N2—C6—C1	122.0 (3)	C33—C32—Fe1	70.2 (2)
C5—C6—C1	119.2 (3)	C31—C32—Fe1	69.2 (2)
N2—C7—C11	123.5 (3)	C33—C32—H32	126.0
N2—C7—C8	125.2 (3)	C31—C32—H32	126.0
C11—C7—C8	111.3 (2)	Fe1—C32—H32	126.0
N3—C8—C7	109.5 (2)	C29—C33—C32	108.0 (4)
N3—C8—C9	114.8 (2)	C29—C33—Fe1	69.3 (2)
C7—C8—C9	100.2 (2)	C32—C33—Fe1	69.7 (2)
N3—C8—C21	103.0 (2)	C29—C33—H33	126.0
C7—C8—C21	111.2 (2)	C32—C33—H33	126.0
C9—C8—C21	118.2 (2)	Fe1—C33—H33	126.0
C15—C9—C10	119.1 (3)	C35—C34—C38	108.1 (3)
C15—C9—C8	129.6 (3)	C35—C34—Fe1	68.91 (19)
C10—C9—C8	111.1 (2)	C38—C34—Fe1	70.42 (17)
C12—C10—C9	121.9 (3)	C35—C34—H34	126.0
C12—C10—C11	129.0 (3)	C38—C34—H34	126.0
C9—C10—C11	109.1 (2)	Fe1—C34—H34	126.0
N1—C11—C7	123.9 (3)	C36—C35—C34	108.4 (3)
N1—C11—C10	128.2 (3)	C36—C35—Fe1	70.2 (2)
C7—C11—C10	107.9 (2)	C34—C35—Fe1	70.14 (18)
C13—C12—C10	118.3 (3)	C36—C35—H35	125.8
C13—C12—H12	120.8	C34—C35—H35	125.8
C10—C12—H12	120.8	Fe1—C35—H35	125.8
C14—C13—C12	120.0 (3)	C35—C36—C37	108.3 (3)
C14—C13—H13	120.0	C35—C36—Fe1	69.5 (2)
C12—C13—H13	120.0	C37—C36—Fe1	70.37 (18)
C13—C14—C15	122.0 (3)	C35—C36—H36	125.8
C13—C14—H14	119.0	C37—C36—H36	125.8
C15—C14—H14	119.0	Fe1—C36—H36	125.8
C9—C15—C14	118.7 (3)	C36—C37—C38	108.1 (3)
C9—C15—H15	120.7	C36—C37—Fe1	68.9 (2)
C14—C15—H15	120.7	C38—C37—Fe1	70.04 (18)
N3—C16—C17	105.6 (2)	C36—C37—H37	125.9
N3—C16—H16A	110.6	C38—C37—H37	125.9
C17—C16—H16A	110.6	Fe1—C37—H37	125.9
N3—C16—H16B	110.6	C37—C38—C34	107.1 (3)
C17—C16—H16B	110.6	C37—C38—C20	127.8 (3)
H16A—C16—H16B	108.8	C34—C38—C20	125.0 (3)
C18—C17—C16	102.0 (3)	C37—C38—Fe1	69.41 (18)
C18—C17—H17A	111.4	C34—C38—Fe1	68.89 (17)
C16—C17—H17A	111.4	C20—C38—Fe1	128.3 (2)
C18—C17—H17B	111.4	C11—N1—C1	114.2 (3)
C16—C17—H17B	111.4	C7—N2—C6	114.5 (3)
H17A—C17—H17B	109.2	C16—N3—C19	108.0 (2)
C17—C18—C19	104.5 (3)	C16—N3—C8	120.4 (2)
C17—C18—H18A	110.9	C19—N3—C8	111.3 (2)
C19—C18—H18A	110.9	C35—Fe1—C31	104.76 (16)
C17—C18—H18B	110.9	C35—Fe1—C30	118.40 (17)
C19—C18—H18B	110.9	C31—Fe1—C30	40.4 (2)
H18A—C18—H18B	108.9	C35—Fe1—C36	40.32 (16)
N3—C19—C18	106.0 (2)	C31—Fe1—C36	120.97 (17)
N3—C19—C20	106.1 (2)	C30—Fe1—C36	105.07 (17)
C18—C19—C20	116.5 (3)	C35—Fe1—C29	155.23 (17)
N3—C19—H19	109.3	C31—Fe1—C29	67.95 (19)
C18—C19—H19	109.3	C30—Fe1—C29	40.78 (19)
C20—C19—H19	109.3	C36—Fe1—C29	121.61 (18)
C38—C20—C19	112.8 (2)	C35—Fe1—C34	40.95 (13)
C38—C20—C21	114.2 (2)	C31—Fe1—C34	120.64 (18)
C19—C20—C21	102.9 (2)	C30—Fe1—C34	154.82 (18)
C38—C20—H20	108.9	C36—Fe1—C34	68.33 (15)
C19—C20—H20	108.9	C29—Fe1—C34	163.11 (16)
C21—C20—H20	108.9	C35—Fe1—C32	123.07 (17)
C22—C21—C20	114.3 (2)	C31—Fe1—C32	40.32 (17)
C22—C21—C8	114.8 (2)	C30—Fe1—C32	67.89 (19)
C20—C21—C8	104.6 (2)	C36—Fe1—C32	158.00 (16)
C22—C21—H21	107.6	C29—Fe1—C32	67.54 (18)
C20—C21—H21	107.6	C34—Fe1—C32	108.66 (16)
C8—C21—H21	107.6	C35—Fe1—C37	68.24 (15)
O1—C22—C23	120.2 (3)	C31—Fe1—C37	158.56 (17)
O1—C22—C21	120.5 (3)	C30—Fe1—C37	123.57 (19)
C23—C22—C21	119.3 (3)	C36—Fe1—C37	40.77 (14)
C28—C23—C24	118.2 (3)	C29—Fe1—C37	109.45 (17)
C28—C23—C22	123.2 (3)	C34—Fe1—C37	68.17 (14)
C24—C23—C22	118.6 (3)	C32—Fe1—C37	160.23 (15)
C25—C24—C23	120.1 (4)	C35—Fe1—C33	160.93 (17)
C25—C24—H24	120.0	C31—Fe1—C33	67.63 (15)
C23—C24—H24	120.0	C30—Fe1—C33	67.94 (17)
C26—C25—C24	119.9 (4)	C36—Fe1—C33	158.65 (17)
C26—C25—H25	120.0	C29—Fe1—C33	40.09 (17)
C24—C25—H25	120.0	C34—Fe1—C33	126.52 (15)
C27—C26—C25	121.1 (4)	C32—Fe1—C33	40.02 (16)
C27—C26—Br1	118.6 (4)	C37—Fe1—C33	125.08 (15)
C25—C26—Br1	120.3 (3)	C35—Fe1—C38	68.70 (13)
C26—C27—C28	119.4 (4)	C31—Fe1—C38	157.77 (19)
C26—C27—H27	120.3	C30—Fe1—C38	161.55 (19)
C28—C27—H27	120.3	C36—Fe1—C38	68.57 (13)
C27—C28—C23	121.3 (4)	C29—Fe1—C38	126.66 (16)
C27—C28—H28	119.4	C34—Fe1—C38	40.69 (13)
C23—C28—H28	119.4	C32—Fe1—C38	124.33 (14)
C33—C29—C30	108.1 (4)	C37—Fe1—C38	40.55 (13)
C33—C29—Fe1	70.6 (2)	C33—Fe1—C38	111.35 (13)
C30—C29—Fe1	69.4 (2)		
			
N1—C1—C2—C3	178.6 (4)	C30—C31—Fe1—C35	−116.7 (3)
C6—C1—C2—C3	−1.2 (7)	C32—C31—Fe1—C35	124.2 (3)
C1—C2—C3—C4	0.1 (8)	C32—C31—Fe1—C30	−119.1 (4)
C2—C3—C4—C5	0.4 (9)	C30—C31—Fe1—C36	−76.3 (3)
C3—C4—C5—C6	0.4 (8)	C32—C31—Fe1—C36	164.6 (2)
C4—C5—C6—N2	179.0 (4)	C30—C31—Fe1—C29	38.3 (3)
C4—C5—C6—C1	−1.6 (6)	C32—C31—Fe1—C29	−80.8 (3)
N1—C1—C6—N2	1.5 (5)	C30—C31—Fe1—C34	−157.9 (2)
C2—C1—C6—N2	−178.6 (3)	C32—C31—Fe1—C34	82.9 (3)
N1—C1—C6—C5	−177.8 (3)	C30—C31—Fe1—C32	119.1 (4)
C2—C1—C6—C5	2.0 (5)	C30—C31—Fe1—C37	−48.9 (5)
N2—C7—C8—N3	−64.2 (4)	C32—C31—Fe1—C37	−168.0 (4)
C11—C7—C8—N3	115.6 (3)	C30—C31—Fe1—C33	81.8 (3)
N2—C7—C8—C9	174.8 (3)	C32—C31—Fe1—C33	−37.4 (3)
C11—C7—C8—C9	−5.5 (3)	C30—C31—Fe1—C38	173.7 (3)
N2—C7—C8—C21	49.0 (4)	C32—C31—Fe1—C38	54.6 (5)
C11—C7—C8—C21	−131.3 (3)	C31—C30—Fe1—C35	79.1 (3)
N3—C8—C9—C15	64.3 (4)	C29—C30—Fe1—C35	−162.5 (3)
C7—C8—C9—C15	−178.5 (3)	C29—C30—Fe1—C31	118.4 (4)
C21—C8—C9—C15	−57.5 (4)	C31—C30—Fe1—C36	120.4 (3)
N3—C8—C9—C10	−110.9 (3)	C29—C30—Fe1—C36	−121.3 (3)
C7—C8—C9—C10	6.3 (3)	C31—C30—Fe1—C29	−118.4 (4)
C21—C8—C9—C10	127.2 (3)	C31—C30—Fe1—C34	49.4 (5)
C15—C9—C10—C12	−1.2 (5)	C29—C30—Fe1—C34	167.8 (3)
C8—C9—C10—C12	174.6 (3)	C31—C30—Fe1—C32	−37.6 (2)
C15—C9—C10—C11	179.2 (3)	C29—C30—Fe1—C32	80.8 (3)
C8—C9—C10—C11	−5.0 (3)	C31—C30—Fe1—C37	160.7 (2)
N2—C7—C11—N1	2.2 (5)	C29—C30—Fe1—C37	−80.9 (3)
C8—C7—C11—N1	−177.5 (3)	C31—C30—Fe1—C33	−80.9 (3)
N2—C7—C11—C10	−177.3 (3)	C29—C30—Fe1—C33	37.4 (3)
C8—C7—C11—C10	2.9 (3)	C31—C30—Fe1—C38	−172.5 (4)
C12—C10—C11—N1	2.3 (6)	C29—C30—Fe1—C38	−54.1 (6)
C9—C10—C11—N1	−178.2 (3)	C37—C36—Fe1—C35	−119.3 (3)
C12—C10—C11—C7	−178.2 (3)	C35—C36—Fe1—C31	−75.6 (3)
C9—C10—C11—C7	1.3 (3)	C37—C36—Fe1—C31	165.1 (2)
C9—C10—C12—C13	0.0 (5)	C35—C36—Fe1—C30	−116.3 (3)
C11—C10—C12—C13	179.5 (3)	C37—C36—Fe1—C30	124.4 (3)
C10—C12—C13—C14	1.0 (5)	C35—C36—Fe1—C29	−157.3 (2)
C12—C13—C14—C15	−0.7 (5)	C37—C36—Fe1—C29	83.4 (3)
C10—C9—C15—C14	1.5 (5)	C35—C36—Fe1—C34	38.0 (2)
C8—C9—C15—C14	−173.4 (3)	C37—C36—Fe1—C34	−81.3 (2)
C13—C14—C15—C9	−0.5 (5)	C35—C36—Fe1—C32	−48.3 (5)
N3—C16—C17—C18	−36.3 (3)	C37—C36—Fe1—C32	−167.6 (4)
C16—C17—C18—C19	35.3 (3)	C35—C36—Fe1—C37	119.3 (3)
C17—C18—C19—N3	−21.9 (3)	C35—C36—Fe1—C33	175.9 (4)
C17—C18—C19—C20	95.8 (3)	C37—C36—Fe1—C33	56.7 (5)
N3—C19—C20—C38	−150.8 (2)	C35—C36—Fe1—C38	81.9 (2)
C18—C19—C20—C38	91.6 (3)	C37—C36—Fe1—C38	−37.4 (2)
N3—C19—C20—C21	−27.1 (3)	C33—C29—Fe1—C35	158.2 (4)
C18—C19—C20—C21	−144.8 (2)	C30—C29—Fe1—C35	39.2 (6)
C38—C20—C21—C22	−76.1 (3)	C33—C29—Fe1—C31	81.0 (3)
C19—C20—C21—C22	161.2 (2)	C30—C29—Fe1—C31	−38.0 (3)
C38—C20—C21—C8	157.6 (2)	C33—C29—Fe1—C30	119.0 (4)
C19—C20—C21—C8	34.9 (3)	C33—C29—Fe1—C36	−165.3 (2)
N3—C8—C21—C22	−155.6 (2)	C30—C29—Fe1—C36	75.8 (3)
C7—C8—C21—C22	87.2 (3)	C33—C29—Fe1—C34	−43.0 (7)
C9—C8—C21—C22	−27.9 (4)	C30—C29—Fe1—C34	−161.9 (5)
N3—C8—C21—C20	−29.5 (3)	C33—C29—Fe1—C32	37.3 (2)
C7—C8—C21—C20	−146.8 (2)	C30—C29—Fe1—C32	−81.7 (3)
C9—C8—C21—C20	98.2 (3)	C33—C29—Fe1—C37	−121.8 (2)
C20—C21—C22—O1	−36.7 (4)	C30—C29—Fe1—C37	119.2 (3)
C8—C21—C22—O1	84.2 (4)	C30—C29—Fe1—C33	−119.0 (4)
C20—C21—C22—C23	142.0 (3)	C33—C29—Fe1—C38	−79.7 (3)
C8—C21—C22—C23	−97.1 (3)	C30—C29—Fe1—C38	161.4 (3)
O1—C22—C23—C28	−179.9 (3)	C38—C34—Fe1—C35	119.3 (3)
C21—C22—C23—C28	1.4 (5)	C35—C34—Fe1—C31	76.6 (3)
O1—C22—C23—C24	0.9 (5)	C38—C34—Fe1—C31	−164.0 (2)
C21—C22—C23—C24	−177.8 (3)	C35—C34—Fe1—C30	41.7 (5)
C28—C23—C24—C25	−0.1 (5)	C38—C34—Fe1—C30	161.1 (4)
C22—C23—C24—C25	179.2 (3)	C35—C34—Fe1—C36	−37.4 (2)
C23—C24—C25—C26	−0.8 (6)	C38—C34—Fe1—C36	81.9 (2)
C24—C25—C26—C27	1.3 (7)	C35—C34—Fe1—C29	−166.7 (6)
C24—C25—C26—Br1	−179.2 (3)	C38—C34—Fe1—C29	−47.3 (6)
C25—C26—C27—C28	−0.8 (6)	C35—C34—Fe1—C32	119.3 (2)
Br1—C26—C27—C28	179.7 (3)	C38—C34—Fe1—C32	−121.3 (2)
C26—C27—C28—C23	−0.1 (6)	C35—C34—Fe1—C37	−81.5 (2)
C24—C23—C28—C27	0.6 (5)	C38—C34—Fe1—C37	37.85 (18)
C22—C23—C28—C27	−178.7 (3)	C35—C34—Fe1—C33	160.2 (2)
C33—C29—C30—C31	−0.3 (5)	C38—C34—Fe1—C33	−80.4 (2)
Fe1—C29—C30—C31	59.9 (3)	C35—C34—Fe1—C38	−119.3 (3)
C33—C29—C30—Fe1	−60.3 (3)	C33—C32—Fe1—C35	168.1 (2)
C29—C30—C31—C32	0.2 (5)	C31—C32—Fe1—C35	−72.7 (3)
Fe1—C30—C31—C32	60.2 (3)	C33—C32—Fe1—C31	−119.2 (4)
C29—C30—C31—Fe1	−60.0 (3)	C33—C32—Fe1—C30	−81.6 (3)
C30—C31—C32—C33	0.0 (4)	C31—C32—Fe1—C30	37.7 (3)
Fe1—C31—C32—C33	59.7 (2)	C33—C32—Fe1—C36	−156.7 (4)
C30—C31—C32—Fe1	−59.8 (3)	C31—C32—Fe1—C36	−37.5 (6)
C30—C29—C33—C32	0.3 (4)	C33—C32—Fe1—C29	−37.3 (2)
Fe1—C29—C33—C32	−59.2 (2)	C31—C32—Fe1—C29	81.9 (3)
C30—C29—C33—Fe1	59.5 (3)	C33—C32—Fe1—C34	125.1 (2)
C31—C32—C33—C29	−0.2 (4)	C31—C32—Fe1—C34	−115.7 (3)
Fe1—C32—C33—C29	58.9 (3)	C33—C32—Fe1—C37	47.8 (6)
C31—C32—C33—Fe1	−59.1 (3)	C31—C32—Fe1—C37	167.1 (4)
C38—C34—C35—C36	0.2 (4)	C31—C32—Fe1—C33	119.2 (4)
Fe1—C34—C35—C36	60.0 (2)	C33—C32—Fe1—C38	82.7 (3)
C38—C34—C35—Fe1	−59.8 (2)	C31—C32—Fe1—C38	−158.1 (3)
C34—C35—C36—C37	0.0 (4)	C36—C37—Fe1—C35	37.4 (2)
Fe1—C35—C36—C37	59.9 (2)	C38—C37—Fe1—C35	−82.2 (2)
C34—C35—C36—Fe1	−60.0 (2)	C36—C37—Fe1—C31	−37.1 (5)
C35—C36—C37—C38	−0.2 (4)	C38—C37—Fe1—C31	−156.8 (4)
Fe1—C36—C37—C38	59.2 (2)	C36—C37—Fe1—C30	−73.0 (3)
C35—C36—C37—Fe1	−59.4 (2)	C38—C37—Fe1—C30	167.3 (2)
C36—C37—C38—C34	0.3 (4)	C38—C37—Fe1—C36	−119.7 (3)
Fe1—C37—C38—C34	58.8 (2)	C36—C37—Fe1—C29	−116.2 (3)
C36—C37—C38—C20	178.4 (3)	C38—C37—Fe1—C29	124.1 (2)
Fe1—C37—C38—C20	−123.1 (3)	C36—C37—Fe1—C34	81.7 (2)
C36—C37—C38—Fe1	−58.5 (2)	C38—C37—Fe1—C34	−37.98 (18)
C35—C34—C38—C37	−0.3 (3)	C36—C37—Fe1—C32	166.2 (4)
Fe1—C34—C38—C37	−59.1 (2)	C38—C37—Fe1—C32	46.6 (5)
C35—C34—C38—C20	−178.5 (3)	C36—C37—Fe1—C33	−158.2 (2)
Fe1—C34—C38—C20	122.7 (3)	C38—C37—Fe1—C33	82.2 (2)
C35—C34—C38—Fe1	58.8 (2)	C36—C37—Fe1—C38	119.7 (3)
C19—C20—C38—C37	152.6 (3)	C29—C33—Fe1—C35	−151.5 (4)
C21—C20—C38—C37	35.4 (4)	C32—C33—Fe1—C35	−32.0 (5)
C19—C20—C38—C34	−29.6 (4)	C29—C33—Fe1—C31	−81.9 (3)
C21—C20—C38—C34	−146.8 (3)	C32—C33—Fe1—C31	37.6 (3)
C19—C20—C38—Fe1	60.0 (3)	C29—C33—Fe1—C30	−38.1 (3)
C21—C20—C38—Fe1	−57.2 (4)	C32—C33—Fe1—C30	81.4 (3)
C7—C11—N1—C1	−1.2 (5)	C29—C33—Fe1—C36	36.5 (5)
C10—C11—N1—C1	178.2 (3)	C32—C33—Fe1—C36	156.0 (4)
C2—C1—N1—C11	179.6 (3)	C32—C33—Fe1—C29	119.5 (3)
C6—C1—N1—C11	−0.5 (5)	C29—C33—Fe1—C34	165.7 (2)
C11—C7—N2—C6	−1.1 (4)	C32—C33—Fe1—C34	−74.8 (3)
C8—C7—N2—C6	178.6 (3)	C29—C33—Fe1—C32	−119.5 (3)
C5—C6—N2—C7	178.7 (3)	C29—C33—Fe1—C37	78.3 (3)
C1—C6—N2—C7	−0.6 (4)	C32—C33—Fe1—C37	−162.2 (2)
C17—C16—N3—C19	23.4 (3)	C29—C33—Fe1—C38	122.1 (3)
C17—C16—N3—C8	−105.9 (3)	C32—C33—Fe1—C38	−118.4 (2)
C18—C19—N3—C16	−0.9 (3)	C37—C38—Fe1—C35	81.0 (2)
C20—C19—N3—C16	−125.3 (3)	C34—C38—Fe1—C35	−37.8 (2)
C18—C19—N3—C8	133.4 (3)	C20—C38—Fe1—C35	−156.4 (3)
C20—C19—N3—C8	8.9 (3)	C37—C38—Fe1—C31	157.6 (4)
C7—C8—N3—C16	−101.0 (3)	C34—C38—Fe1—C31	38.8 (4)
C9—C8—N3—C16	10.7 (4)	C20—C38—Fe1—C31	−79.8 (5)
C21—C8—N3—C16	140.6 (3)	C37—C38—Fe1—C30	−35.4 (5)
C7—C8—N3—C19	131.2 (2)	C34—C38—Fe1—C30	−154.2 (5)
C9—C8—N3—C19	−117.1 (3)	C20—C38—Fe1—C30	87.2 (6)
C21—C8—N3—C19	12.8 (3)	C37—C38—Fe1—C36	37.6 (2)
C36—C35—Fe1—C31	120.8 (3)	C34—C38—Fe1—C36	−81.3 (2)
C34—C35—Fe1—C31	−120.0 (3)	C20—C38—Fe1—C36	160.1 (3)
C36—C35—Fe1—C30	79.6 (3)	C37—C38—Fe1—C29	−76.6 (3)
C34—C35—Fe1—C30	−161.2 (3)	C34—C38—Fe1—C29	164.6 (2)
C34—C35—Fe1—C36	119.2 (3)	C20—C38—Fe1—C29	45.9 (4)
C36—C35—Fe1—C29	51.6 (5)	C37—C38—Fe1—C34	118.8 (3)
C34—C35—Fe1—C29	170.8 (4)	C20—C38—Fe1—C34	−118.6 (4)
C36—C35—Fe1—C34	−119.2 (3)	C37—C38—Fe1—C32	−162.7 (2)
C36—C35—Fe1—C32	160.5 (2)	C34—C38—Fe1—C32	78.5 (2)
C34—C35—Fe1—C32	−80.3 (3)	C20—C38—Fe1—C32	−40.2 (3)
C36—C35—Fe1—C37	−37.8 (2)	C34—C38—Fe1—C37	−118.8 (3)
C34—C35—Fe1—C37	81.3 (2)	C20—C38—Fe1—C37	122.5 (4)
C36—C35—Fe1—C33	−175.5 (4)	C37—C38—Fe1—C33	−119.5 (2)
C34—C35—Fe1—C33	−56.3 (5)	C34—C38—Fe1—C33	121.7 (2)
C36—C35—Fe1—C38	−81.6 (2)	C20—C38—Fe1—C33	3.1 (3)
C34—C35—Fe1—C38	37.6 (2)		

### Hydrogen-bond geometry (Å, º)

Cg1, Cg2 and Cg3 are the centroids of the C29–C33, C9–C15 and N1/N2/C1/C6/C7/C11 rings, respectively.

**Table:**

*D*—H···*A*	*D*—H	H···*A*	*D*···*A*	*D*—H···*A*
C28—H28···N2	0.93	2.56	3.358 (5)	145
C13—H13···O1^i^	0.93	2.58	3.318 (4)	137
C4—H4···*Cg*1^ii^	0.93	2.69	3.580 (4)	159
C17—H17*B*···*Cg*2^iii^	0.97	2.84	3.743 (4)	155
C36—H36···*Cg*3^iv^	0.98	2.70	3.486 (5)	137

Symmetry codes: (i) −*x*+1, −*y*, −*z*+2; (ii) −*x*+1, −*y*+1, −*z*+1; (iii) −*x*+1, −*y*+1, −*z*+2; (iv) *x*−1, *y*, *z*.

## References

[bb1] Bernstein, J., Davis, R. E., Shimoni, L. & Chang, N.-L. (1995). *Angew. Chem. Int. Ed. Engl.* **34**, 1555–1573.

[bb2] Biot, C., Dessolin, J., Richard, I. & Dive, D. (2004). *J. Organomet. Chem* **689**, 4678–4682.

[bb3] Bruker (2008). *APEX2*, *SAINT* and *SADABS* Bruker AXS Inc., Madison, Wisconsin, USA.

[bb4] Farrugia, L. J. (2012). *J. Appl. Cryst.* **45**, 849–854.

[bb5] Fouda, M. F. R., Abd-Elzaher, M. M., Abdelsamaia, R. A. & Labib, A. A. (2007). *Appl. Organomet. Chem* **21**, 613–625.

[bb6] Jaouen, G., Top, S., Vessireres, A., Leclercq, G., Vaissermann, J. & McGlinchey, M. J. (2004). *Curr. Med. Chem* **11**, 2505–2517. 10.2174/0929867043364487 15379709

[bb7] Sheldrick, G. M. (2008). *Acta Cryst.* A**64**, 112–122. 10.1107/S0108767307043930 18156677

[bb8] Spek, A. L. (2009). *Acta Cryst.* D**65**, 148–155. 10.1107/S090744490804362X PMC2631630 19171970

[bb9] Vijayakumar, B., Gavaskar, D., Srinivasan, T., Raghunathan, R. & Velmurugan, D. (2012). *Acta Cryst.* E**68**, m1382–m1383. 10.1107/S1600536812042468 PMC3515131 23284358

